# Intimate partner violence and poor mental health among Thai women residing in Sweden

**DOI:** 10.3402/gha.v7.24991

**Published:** 2014-09-16

**Authors:** Cecilia Fernbrant, Maria Emmelin, Birgitta Essén, Per-Olof Östergren, Elizabeth Cantor-Graae

**Affiliations:** 1 Department of Clinical Sciences Malmo, Social Medicine and Global Health, Lund University, Lund, Sweden; 2 Department of Women's and Children's Health, International Maternal and Child Health, Uppsala University, Uppsala, Sweden

**Keywords:** intimate partner violence, poor mental health, social isolation, social capital, Thai women, migration

## Abstract

**Objectives:**

The current aim is to examine the prevalence of intimate partner violence (IPV) among Thai women residing in Sweden and its association with mental health. We also investigate the potential influence of social isolation and social capital regarding the association between IPV and mental health outcome.

**Design:**

A public health questionnaire in Thai was distributed by post to the entire population of Thai women, aged 18–64, residing in two regions in Sweden since 2006. Items included aspects related to IPV (physical/sexual/emotional), sociodemographic background, physical health, mental health (GHQ-12), social isolation, and social capital (i.e. social trust/participation).

**Results:**

The response rate was 62.3% (*n*=804). Prevalence of lifetime reported IPV was 22.1%, with 20.5% by a previous partner and 6.7% by a current partner. Previous IPV exposure was significantly related to current IPV exposure, and all IPV exposure measures were significantly related to poor mental health. However, Thai women experiencing IPV by a current partner were more at risk for poor mental health than Thai women with previous or without any experience of IPV. Also, among all women exposed to IPV, those with trust in others and without exposure to social isolation seemed to have partial protection against the adverse mental health consequences associated with IPV.

**Conclusions:**

Most Thai women had never been exposed to IPV, and after migrating to Sweden, women had lower IPV exposure than in Thailand. However, the increased risk for poor mental health among those Thai women exposed to IPV suggests the need for supportive measures and targeted interventions to prevent further injuries and adverse health consequences. Although poor mental health in Thai women represents an obstacle for integration, the potential resilience indicated in the group with high social trust and without exposure to social isolation suggests that such aspects be included in the program designed to facilitate integration.

Intimate partner violence (IPV) is the most common type of violence against women and a major public health concern (1–3). IPV is associated with a wide variety of adverse health outcomes, including somatic (2, 4) and mental health disorders (4, 5), and suicide (5), with mortality due to IPV representing the most extreme adverse outcome (6).

Uneducated and less empowered women, for example, women economically dependent on their husbands, are at greater risk of IPV [e.g. (7)]. Thus, some foreign-born women and especially those migrating from low-income countries and countries with low gender equity may potentially be at increased risk of violence in the home (8), and mortality due to violence (9). Factors contributing to their increased risk include patriarchal structures sanctioning women's subordination to men (10), shifts in the relationship's power balance after migration (11), social isolation (12), and low social participation in the new country due to language barriers and lack of social networks (13).

Research concerning IPV exposure among Thai women residing abroad is scarce. However, Thailand was one of the sites included in the WHO multi-country study on women's health and domestic violence against women (14). The results showed that the prevalence of current exposure to physical and/or sexual violence was approximately 22.1% (14). Thus, some Thai women who migrate abroad may previously have been exposed to IPV in Thailand. Foreign migration among Thai women is often due to family reasons, such as marriage and partnerships with persons residing abroad.

Previous research has shown that exposure to both emotional IPV (15) and physical and/or sexual IPV (1, 2, 16) are risk factors for poor mental health. The most common mental health disorders associated with IPV are depression [e.g. (16, 17)] and posttraumatic stress disorder (5). Results from Ellsberg et al.'s WHO's multi-country study (2) showed that Thai women with lifetime exposure to IPV had poorer mental health than unexposed women. Also, the study showed an association between exposure to IPV and suicide attempts, which is supported by previous research (5, 15). Exposure to IPV is also related to poor mental health among foreign-born women more generally (17, 18).

Little is known about the health, well-being, and exposure to IPV among Thai women residing in Sweden. However, informal contact with the Thai community in southern Sweden indicated that both social isolation and IPV were perceived as problems experienced by and relevant for Thai women. According to Swedish legislation, Thai women who are married to a Swedish resident receive a 2-year temporary permit of residence, after which a permanent residence may be given if they are still married. During this period, the woman's main source of economic support is her partner, that is, the Swedish resident, in contrast to, for example, refugees who can get support from Social Services upon arrival. In addition, it is difficult for newly arrived Thai women, who do not speak Swedish, to get a job. Thus, due to lack of employment and poor language skills, social isolation is also a risk factor for poor mental health, especially among foreign-born women (19, 20).

In addition to social isolation, social capital is increasingly regarded as an important determinant of health and well-being. Although social capital has been defined in various ways, the focus in the current study is on social participation and social trust, measures that are frequently used as markers of social capital (21). Low social participation and low social trust have been shown to be associated with poor somatic and mental health [e.g. (22)]. A previous study conducted in Thailand showed that low social trust and low social support were associated with low self-rated health (23), albeit no such research exists concerning Thai women living in Sweden. Although high levels of empowerment, for example, employment, literacy, are associated with less exposure to IPV [e.g. (10)], relatively little is known concerning the potential protective aspects of social capital with regard to the adverse health consequences of IPV [but see (24)]. It could be hypothesized that social capital may act as a buffering mechanism with regard to adverse health consequences of IPV.

To our knowledge, no previous research exists concerning IPV among Thai women residing in Sweden. Thus, the aim of the current study is to examine the prevalence of IPV among Thai women residing in Sweden and its association with mental health. A further aim is to investigate the potential influence of social isolation and social capital measures with regard to the association between IPV and mental health outcome.

## Material and methods

### Study population

This cross-sectional study of Thai women's health is based on self-reported information derived from a postal questionnaire in Thai, sent to the entire population of Thai women aged 18–64 (*n*=1,291) in two regions (Skåne and Sjuhäradsbygden), with residence in Sweden since 2006. These two areas were chosen as part of an initiative from the participating municipalities concerning risk for marginalization among Thai women. The age range was chosen in order to examine women in the work force. All women received three postal reminders to ensure satisfactory response rate. Respondents were informed that their answers would remain anonymous. The final study population consisted of 804 Thai women aged 18–61. The response rate was thus 62.3% of the total target population sample (*n*=1,291). The Lund University Ethical Review Board, dnr 2011/521, granted approval for the study.

### Study setting

The current study was conducted in two regions: Skåne and Sjuhäradsbygden. The former is the most southern region in Sweden, with 33 municipalities comprising approximately 1,260,000 inhabitants, of whom 18% are foreign-born. Malmö (300,000 inhabitants) is the largest city in the region and also the third largest city in the country. Sjuhäradsbygden is situated in the southwest of Sweden and consists of seven municipalities. The region has approximately 185,000 inhabitants of whom 16% are foreign-born.

### Questionnaire

The questionnaire contained 98 questions pertaining to health-related issues, such as self-rated and mental health, disease, exposure to violence, alcohol use, smoking, physical exercise, living conditions, social participation, and other background factors, including marital status, employment, and socioeconomic status. Questionnaire items were based on previous public health surveys in Sweden and elsewhere, for example, the Skåne Public Health Survey (25). Respondents chose their answers among several structured alternatives. The questionnaire was pre-tested in focus group discussions with Thai women to ensure cultural relevance. The questionnaire was constructed in Swedish, translated into Thai, and then independently back translated into Swedish.

### Outcome measure

Poor mental health was selected as the outcome measure. Mental health was assessed by the 12-item General Health Questionnaire (26), a widely used and validated questionnaire evaluating the subjective well-being (in the past 4 weeks), with focus on depression and anxiety. The GHQ has shown cross-cultural validity in a variety of international settings (27), although no validation studies have been conducted in Thailand or among Thai immigrants. The GHQ has four answer alternatives for each item: ‘better than usual’, ‘same as usual’, ‘worse than usual’, ‘much worse than usual’. For the purpose of obtaining a summary score, answer alternatives were aggregated as 1=low (worse than usual/much worse than usual) and 0=high (better than usual/same as usual). Thereafter, a mean summary score was calculated in order to designate women having ‘poor’ versus ‘good’ mental health. For the purpose of data analysis, ‘poor’ mental health was defined as a mean summary score of 2 or more, based on an examination of the frequency distribution, whereby these scores represented the upper quartile. Moreover, this cutoff point corresponds to the sample mean, which is generally recommended as the appropriate cutoff point (27).

### Explanatory measures

Possible determinants of poor mental health were selected by reviewing the literature and included the following: 1) exposure to IPV, 2) demographic and socioeconomic variables, and, 3) self-reported health, social isolation, and social capital measures represented by social trust and social participation.

#### Exposure to IPV

Exposure to insulting/humiliating behavior and/or threat of IPV, defined here as exposure to emotional IPV, and exposure to physical and/or sexual IPV, defined here as physical/sexual IPV, were based on the responses to four questions developed for the purpose of the study. Three questions were derived from selected items used in the WHO's multi-country study (questions 1, 3, 4) and one question, that is, question 2, was derived from an item in the previous Skåne public health survey (25). The four questions were: 1) ‘Have you ever been insulted or humiliated by anyone?’, 2) ‘Have you ever been exposed to threat of violence to the extent that you became frightened?’, 3) ‘Have you ever been slapped, kicked, or in any other way exposed to physical violence?’, and 4) ‘Have you ever been forced to have sexual intercourse or other sexual activity against your will?’ Possible answers were ‘yes’ and ‘no’ to all four questions. The respondents were also asked to select the perpetrator of the violence, according to the following categories: ‘current partner’, ‘previous partner’, ‘other person in the family’, and ‘other person’, as well as the country where such violence occurred: ‘Thailand’ and/or ‘Sweden’ and/or ‘other country’. The current study solely concerns IPV, that is, by current or previous partner, whether in Sweden, Thailand, or elsewhere, and excludes other types of interpersonal violence. Due to the relatively few reports of physical/sexual IPV, an aggregate variable consisting of both physical/sexual IPV and emotional IPV was constructed for the purpose of multivariate analysis.

#### Demographic and socioeconomic variables

The study population of 804 Thai women aged 18–61 was stratified into two age groups, that is, 15–29 and 30–61, based on previous studies showing that women under the age of 30 are more at risk of IPV than older women (14). Marital status was dichotomized as unmarried/divorced/widow or married/cohabitation/partnered. The socioeconomic measures were educational level, employment status, and disposable income. Educational level was divided into two groups, that is, low/middle (≤9 years), high (>9 years) based on the median of the total sample, and employment status was categorized as working full-time or part-time versus unemployed. Disposable income was defined as the woman's monthly disposable income, and dichotomized as: high (≥9,000 SEK≈≥1,300 US$) and low (<9,000 SEK≈<1,300 US$). Immigration year was defined as the year the woman received residence permit in Sweden and stratified into two groups: years 2006–2008 and 2009–2011. The stratification was based on the Swedish legislation regarding family immigration of a third country citizen. Thus, women with residence between the years 2009 and 2011 would not yet be eligible for permanent residence.

#### Self-rated health, social isolation, and social capital measures

Self-rated health was assessed by the question ‘How do you rate your general health status?’ and dichotomized as ‘neither good nor bad/bad/very bad health’ (poor self-rated health) and ‘good/very good health’ (28). Social isolation was based on the response to the question: ‘Have there been periods during the past year when you have felt isolated or excluded from the society?’ Possible answers were ‘yes’ and ‘no’. Social capital was operationalized in terms of social participation and social trust, respectively. Social participation was based on reported participation in the activities of formal or informal groups and was assessed by 13 questions regarding participation in activities during the past year such as meeting of Thai organizations, meeting of other organizations, religious event, sports event, or private party. Based on an examination of the frequency distribution, women with attendance at ≤2 of the activities were considered to have low social participation and women with attendance at ≥3 of the activities were considered to have medium/high participation. Social trust was based on the responses to four statements on general interpersonal trust, namely 1) ‘Most people would take advantage of you if they got the chance’, 2) ‘Most people try to be fair’, 3) ‘You can trust most people’, and 4) ‘You cannot be too careful in dealing with other people’, with four potential responses ranging from ‘Agree completely’ to ‘Do not agree at all’. Social trust was dichotomized, with low social trust defined as agreeing completely/agreeing to questions 1 and 4 and disagreeing completely/disagreeing to questions 2 and 3. The questions concerning social participation and social trust have previously been used in Swedish population surveys [e.g. (25)].

### Data and statistical analysis

Statistical calculations were carried out using SPSS computer software for Macintosh 20.0. Descriptive statistics were used to calculate mean age. The relationship between IPV and poor mental health and demographic and socioeconomic background characteristics, self-rated health, social isolation, and social capital measures, respectively, was analyzed using bivariate logistic regressions, with 95% confidence intervals, in order to calculate crude odds ratios to estimate associations. Multivariate logistic regression analyses were used to calculate odds ratios with 95% confidence intervals for the outcome measure, that is, poor mental health, in relation to exposure to IPV by a current partner, adjusted stepwise for the potential confounders represented by IPV by a previous partner; the demographic and socioeconomic factors represented by age, marital status, educational level, and disposable monthly income; and the measures social isolation, social participation, social trust, and social capital. We chose disposable income level as a better measure for the women's socioeconomic status than employment status, due to their recent duration of residence in Sweden. Self-rated health was not included in the model as it was regarded as a potential mediator between IPV exposure and poor mental health, rather than a confounder. An effect modification analysis was also performed in order to examine the potential combined effect of IPV exposure by a current partner and social isolation, and that of IPV by a current partner and low social trust, both with regard to poor mental health. Statistical significance was set at *p*<0.05.

## Results

The distribution of demographic and socioeconomic background characteristics, self-reported health measures including exposure to IPV and social isolation, social participation, and social trust are presented in Table 1. The mean age was 37, with 82.8% of the women in the older age group (30–61). Of the total sample, 85.4% were married or cohabiting (Table 1), and approximately 75% of these women who were married/cohabiting had a Swedish partner (data not shown). More than half of the women (52.1%) had low or middle educational level, 39.3% worked full-time or part-time, and 83.3% had low disposable income. One-third of the women had resided in Sweden for less than 2 years. One-third also reported poor self-rated health and 19.8% reported poor mental health. Finally, 39.9% reported being socially isolated, almost as many reported low social trust, and half of the women had low social participation (Table 1).

**Table 1:** Background characteristics, health-related measures, social isolation, and social capital measures in Thai women (*n*=804)^a^

		*N* (%)
Age	18–29	177 (17.2)
	30–61	627 (82.8)
	Total	804
Married/cohabiting	Yes	618 (85.4)
	No	106 (14.6)
	Total	724
Educational level	0–9 years	392 (52.1)
	≥10 years	361 (47.9)
	Total	753
Employment^b^	Yes	316 (39.3)
	No	488 (60.7)
	Total	804
Disposable income^c^	<9,000	670 (83.3)
	≥9,000	134 (16.7)
	Total	804
Immigration year	2006–2008	554 (68.9)
	2009–2011	250 (31.1)
	Total	804
Self-rated health	Poor	223 (27.9)
	Good	575 (72.1)
	Total	798
Mental health^d^	Poor	152 (19.8)
	Good	617 (80.2)
	Total	769
Social isolation	Yes	297 (39.9)
	No	447 (60.1)
	Total	744
Low social participation^e^	Yes	388 (48.3)
	No	416 (51.7)
	Total	804
Low social trust	Yes	262 (37.6)
	No	435 (62.4)
	Total	697

a Mean age=37.34; median age=37.00; std. deviation=8.64; quartiles=25: 31.00, 50: 37.00, 75: 43.00.

b Employed full-time or part-time.

c Disposable monthly household income: ≥9000 SEK≈≥1.300 US$,<9,000 SEK≈<1.300 US$.

d Poor mental health (GHQ 12): ≥2 items of 12.

e Low social participation:≤2 activities of 13.

Table 2 shows information concerning IPV prevalence, perpetrator, and IPV-related injuries in the study population. In the total sample (*n*=804), 178 (22.1%) of the women reported lifetime exposure to IPV (emotional and/or physical and/or sexual), that is, either by previous or current partner, in Sweden or elsewhere, and 74 (9.2%) had lifetime exposure to IPV since they moved to Sweden (Table 2). Of these 178 women, 41 (23.0%) had been exposed to IPV, regardless of type, both by a previous and a current partner (data not shown). In addition, current exposure to IPV was strongly related to exposure to IPV by a previous partner (OR, 15.92; 95% CI, 8.29–30.59, data not shown). However, the majority of women with IPV exposure in Thailand (*n*=133) did not have IPV exposure in Sweden (data not shown).

**Table 2:** Prevalence and perpetrator of intimate partner violence and intimate partner violence–related injuries in Thai women

		*N* (%)
IPV ever in lifetime	Yes	178 (22.1)
	No	626 (77.9)
Emotional IPV	Yes	128 (15.9)
	No	676 (84.1)
Physical/sexual IPV	Yes	119 (14.8)
	No	685 (85.2)
IPV by previous partner^a^	Yes	165 (20.5)
	No	639 (79.5)
Emotional IPV	Yes	115 (14.3)
	No	689 (85.7)
Physical/sexual IPV	Yes	112 (13.9)
	No	692 (86.1)
IPV by current partner^b^	Yes	54 (6.7)
	No	750 (93.3)
Emotional IPV	Yes	49 (6.1)
	No	755 (93.9)
Physical/sexual IPV	Yes	19 (2.4)
	No	785 (97.6)
IPV in Thailand^c^	Yes	133 (16.5)
	No	671 (83.5)
IPV in Sweden^c^	Yes	74 (9.2)
	No	730 (90.8)
IPV elsewhere^c^	Yes	11 (1.4)
	No	793 (98.6)
Injuries due to physical/sexual IPV	Yes	35 (35.4)
	No	64 (64.6)
	Total	99
Sought health care	Yes	16 (38.1)
	No	26 (61.9)
	Total	42

a 124 of these were solely exposed by a previous partner.

b 13 of these were solely exposed by a current partner.

c Any type of IPV, that is, emotional, physical, or sexual.

Exposure to IPV by a previous partner was reported by 165 women (20.5%), with approximately equivalent rates for emotional IPV and physical/sexual IPV, and 54 (6.7%) of the women reported IPV by a current partner, with emotional IPV more frequent than physical/sexual IPV (Table 2). The majority of women with current IPV exposure had a Swedish partner. Among the women who had been exposed to physical and/or sexual IPV, whether by a previous or a current partner, 35.4% reported physical and/or psychological injuries, and 38.1% had sought health care for their injuries (Table 2). Also, 25.7% of those who had been exposed to any type of IPV had told someone about the abuse, most often a close friend or relative (data not shown).

Table 3 shows the relationship between IPV by current partner and demographic and socioeconomic background characteristics, social isolation, and social capital measures. Odds ratios for IPV were not significantly elevated for any of the background characteristics. With regard to the social variables, the odds ratio for IPV exposure was significantly elevated solely in relation to social isolation (OR, 3.37; 95% CI, 1.82–6.24) (Table 3). With regard to IPV by previous partner, similar non-significant results were obtained in relation to background characteristics, but odds ratios for IPV in relation to social isolation (OR, 2.17; 95% CI, 1.50–3.13) and low social trust (OR, 1.58; 95% CI, 1.08–2.30) were nevertheless significant (data not shown).

**Table 3:** Intimate partner violence by a current partner in relation to background characteristics, social isolation, and social capital measures in Thai women

		IPV current partner^a^		
		
		Yes	No	Crude OR
		*N* (%)	*N* (%)	OR (95% CI)
Age	18–29	5 (9.3)	133 (17.7)	0.47 (0.18–1.21)
	30–61	49 (90.7)	617 (82.3)	1
Married/cohabiting	Yes	38 (84.4)	580 (85.4)	1
	No	7 (15.6)	99 (14.6)	1.08 (0.47–2.49)
Educational level	0–9 years	30 (63.8)	362 (51.3)	1.68 (0.91–3.10)
	>9 years	17 (36.2)	344 (48.7)	1
Employment^b^	Yes	22 (40.7)	294 (39.2)	1
	No	32 (59.3)	456 (60.8)	0.94 (0.53–1.65)
Disposable income^c^	<9,000	45 (83.3)	625 (83.3)	1.00 (0.48–2.10)
	≥9,000	9 (16.7)	125 (16.7)	1
Immigration year	2006–2008	39 (72.2)	515 (68.7)	1
	2009–2011	15 (27.8)	235 (31.3)	0.84 (0.46–1.56)
Socially isolated	No	16 (32.7)	431 (62.0)	1
	Yes	33 (67.3)	264 (38.0)	**3.37 (1.82–6.24)** e
Low social participation^d^	No	21 (38.9)	395 (52.7)	1
	Yes	33 (61.1)	355 (47.3)	1.75 (0.99–3.08)
Low social trust	No	22 (50.0)	413 (63.2)	1
	Yes	22 (50.0)	240 (36.8)	1.72 (0.93–3.17)

a IPV exposure regarding current and previous partner includes 41 women with experiences of both.

b Employed full or part-time.

c Disposable monthly household income: ≥9,000 SEK≈≥1.300 US$, <9,000 SEK≈<1.300 US$.

d Low social participation:≤2 activities of 13.

e Bold values show statistical significance at p<0.05.

Table 4 shows the relationship between self-reported mental health and exposure to IPV, demographic and socioeconomic background characteristics, self-rated health, social isolation, and social capital measures. Thai women who reported poor mental health had significantly elevated odds ratios for all types of IPV. Odds ratios for poor mental health were greater among women with current partner abuse than for those with previous partner abuse, regardless of abuse type.

**Table 4:** Poor mental health in relation to background characteristics, intimate partner violence^a^, self-rated health, social isolation, and social capital measures in Thai women

		Yes	No	Crude OR
Poor mental health^b^		*N* (%)	*N* (%)	OR (95% CI)
Emotional IPV by previous partner	No	115 (75.7)	543 (88.0)	1
	Yes	37 (24.3)	74 (12.0)	**2.36 (1.52–3.68)** f
Physical/sexual IPV by previous partner	No	121 (79.6)	542 (87.8)	1
	Yes	31 (20.4)	75 (12.2)	**1.85 (1.17–2.94)** f
Emotional IPV by current partner	No	129 (84.9)	596 (96.6)	1
	Yes	23 (15.1)	21 (3.4)	**5.06 (2.72–9.42)** f
Physical/sexual IPV by current partner	No	142 (97.7)	609 (98.7)	1
	Yes	10 (6.6)	8 (1.3)	**5.36 (2.08–13.83)** f
Age	18–29 years	29 (19.1)	108 (17.5)	1.11 (0.71–1.75)
	30–61 years	123 (80.9)	509 (82.5)	1
Married/cohabiting	Yes	109 (79.0)	487 (87.0)	1
	No	29 (21.0)	73 (13.0)	**1.78 (1.10–2.86)** f
Educational level	0–9 years	64 (47.1)	298 (51.1)	0.85 (0.59–1.24)
	>9 years	72 (52.9)	285 (48.9)	1
Employment status^c^	Yes	55 (36.2)	246 (39.9)	1
	No	97 (63.8)	371 (60.1)	1.17 (0.81–1.69)
Disposable income^d^	<9,000	127 (83.6)	518 (84.0)	0.97 (0.60–1.57)
	≥9,000	25 (16.4)	99 (16.0)	1
Immigration year	2006–2008	106 (69.7)	418 (67.7)	1
	2009–2011	46 (30.3)	199 (32.3)	0.91 (0.62–1.34)
Self-rated health	Good	69 (46.0)	484 (78.6)	1
	Poor	81 (54.0)	132 (21.4)	**4.30 (2.96–6.26)** f
Socially isolated	No	49 (33.8)	382 (66.8)	1
	Yes	96 (66.2)	190 (33.2)	**3.94 (2.68–5.79)** f
Low social participation^e^	No	69 (45.4)	335 (54.3)	1
	Yes	83 (54.6)	282 (45.7)	1.43 (0.99–2.04)
Low social trust	No	57 (42.2)	366 (67.5)	1
	Yes	78 (57.8)	176 (32.5)	**2.85 (1.93–4.19)** f

a IPV exposure regarding previous and current partner includes 41 women with experiences of both.

b Poor mental health (GHQ 12):≥2 items of 12.

c Employed full-time or part-time.

d Disposable monthly household income:≥9,000 SEK≈≥1.300 US$,<9,000 SEK≈<1.300 US$.

e Low social participation:≤2 activities of 13.

f Bold values show statistical significance at p<0.05.

No significant relationship was observed between mental health and demographic and socioeconomic background characteristics, except for poorer mental health among women who were not in a relationship (OR, 1.78; 95% CI, 1.10–2.86). Finally, odds ratios for poor mental health was significantly elevated in women with poorer self-rated health (OR, 4.30; 95% CI, 2.96–6.26), perceived social isolation (OR, 3.94; 95% CI, 2.68–5.79), and lower social trust (OR, 2.85; 95% CI, 1.93–4.19), versus women with good mental health (Table 4).

As current IPV exposure was more strongly related to poor mental health than previous IPV exposure, a multivariate logistic regression analysis was performed in order to examine the relationship between poor mental health and IPV exposure by a current partner, with non-exposure to IPV as the reference category (Table 5). In the crude model, women exposed to IPV by a current partner had significantly elevated odds ratios for poor mental health (OR, 4.86; 95% CI, 2.69–8.79), compared to unexposed women. In the fully adjusted model, the increased odds of poor mental health among women exposed to IPV by a current partner became somewhat attenuated but remained significant (OR, 3.29; 95% CI, 1.39–7.79) after stepwise adjustment for the potential confounders represented by IPV exposure by a previous partner, age, marital status, educational level, disposable income, social isolation, and social capital measures (Table 5). In the fully adjusted model, perceived social isolation (OR, 5.15; 95% CI, 3.18–8.33), and low social trust (OR, 2.43; 95% CI, 1.53–3.85) were also significantly related to poor mental health. Exposure to IPV by a previous partner was not significantly associated with poor mental health in the multivariate analysis. Multivariate analyses performed separately for the measures emotional and physical/sexual IPV, respectively, showed similar but non-significant relationships as and when the aggregate variable was used.

**Table 5:** Poor mental health in relation to intimate partner violence among Thai women

	Crude OR	Model 1	Model 2	Model 3
	OR (95% CI)	OR (95% CI)	OR (95% CI)	OR (95% CI)
IPV current partner^a^	**4.86 (2.69–8.79)** j	**4.13 (2.14–7.97)** j	**3.23 (1.57–6.65)** j	**3.29 (1.39–7.79)** j
IPV previous partner^b^		1.30 (0.82–2.07)	1.30 (0.79–2.16)	0.88 (0.49–1.58)
Age^c^			1.09 (0.65–1.82)	1.24 (0.68–2.25)
Marital status^d^			**1.81 (1.07–3.04)** j	1.74 (0.94–3.20)
Low education^e^			0.77 (0.51–1.14)	0.88 (0.55–1.42)
Disposable income^f^			1.22 (0.70–2.15)	0.80 (0.43–1.51)
Social isolation^g^				**5.15 (3.18–8.33)** j
Low social participation^h^				1.35 (0.85–2.15)
Low social trust^i^				**2.43 (1.53–3.85)** j

a Emotional and/or physical and/or sexual IPV: yes, no (ref.).

b Emotional and/or physical and/or sexual IPV: yes, no (ref.).

c Age groups: 18–29, 30–61 (ref.).

d Married/cohabitation/partnered, unmarried/divorced/widow (ref.).

e Educational level: low/medium≤9 years, high>9years (ref.).

f Disposable monthly income:≤9,000 SEK,>9,000 SEK (ref.).

g Social isolation: yes, no (ref.).

h Low social participation: yes, no (ref.).

i Low social trust: yes, no (ref.).

j Bold values show statistical significance at p<0.05.

The results of the effect modification analysis showed a synergy effect between IPV by a current partner and social isolation with regard to the outcome measure of poor mental health (*p*<0.001, fully adjusted model, Table 6). The lower odds ratios for women exposed to IPV but who were not socially isolated indirectly indicate a greater resilience to adverse mental health consequences of IPV. Similarly, a synergy effect was found between IPV exposure by a current partner and low social trust (*p*<0.001, fully adjusted model, Table 6). The lower odds ratios for women exposed to IPV with high social trust indirectly indicate greater resilience to adverse mental health consequences of IPV. No such synergy effect was obtained for social participation.

**Table 6:** Effect modification analysis between intimate partner violence (IPV) by a current partner^a^ and social isolation^b^, social participation^c^, and social trust^d^, respectively, with regard to poor mental health^e^ in Thai women

	Poor mental health		
	
	Yes	No	Crude OR
	*N* (%)	*N* (%)	OR (95% CI)
No IPV, not socially isolated	43 (29.7)	373 (65.2)	1
No IPV, socially isolated	77 (53.1)	179 (31.3)	**3.73 (2.47–5.64)** f
IPV, not socially isolated	6 (4.1)	9 (1.6)	**5.78 (1.96–17.03)** f
IPV, socially isolated	19 (13.1)	11 (1.9)	**14.98 (6.69–33.58)** f
No IPV, high social participation	56 (36.8)	328 (53.2)	1
No IPV, low social participation	71 (46.7)	265 (42.9)	**1.89 (1.24–2.91)** f
IPV, high social participation	13 (8.6)	7 (1.1)	**6.94 (2.23–20.71)** f
IPV, low social participation	12 (7.9)	17 (2.8)	**3.44 (1.34–8.80)** f
No IPV, high social trust	48 (35.6)	355 (65.5)	1
No IPV, low social trust	65 (48.1)	168 (31.0)	**2.86 (1.89–4.34)** f
IPV, high social trust	9 (6.7)	11 (2.0)	**6.05 (2.39–15.35)** f
IPV, low social trust	13 (9.6)	8 (1.5)	**12.02 (4.74–30.49)** f

a Emotional and/or physical and/or sexual IPV: yes, no (ref.).

b Social isolation: yes, no (ref.).

c Social participation: yes, no (ref.).

d Low social trust: yes, no (ref.).

e Poor mental health (GHQ 12):≥2 items of 12.

f Bold values show statistical significance at p<0.05.

## Discussion

In this sample of Thai women residing in Sweden, 22.1% reported lifetime exposure to IPV and 9.2% had been exposed to IPV since they moved to Sweden. Thus, most of the women had not been exposed to IPV. However, all types of IPV both by a current and/or a previous partner, that is, emotional, and/or physical, and/or sexual IPV, were significantly related to poor mental health. In the fully adjusted model, the association between exposure to IPV by a current partner and poor mental health remained significant, albeit exposure to IPV by a previous partner was no longer significantly associated with poor mental health. Moreover, in the fully adjusted model, social isolation and low social trust were also independently associated with poor mental health. Among women exposed to IPV, those without social isolation and those with high social trust appeared to have greater resilience with regard to adverse mental health consequences of IPV, compared to socially isolated women and women with low social trust.

It should be noted that IPV by a current partner was rather low (6.7%) compared to previous experiences of abuse (20.5%), and most of the women in this sample had never been exposed to abuse. However, most of the currently abused women had previous experience of IPV exposure, and such exposure was also strongly related to IPV exposure by a current partner. These results corroborate findings from other studies showing that previous experience of IPV is a risk factor for such violence, as well as for mortality due to IPV (6). Of the women with exposure to IPV by a current partner, the majority of the partners originated from Sweden. Rates of current physical/sexual IPV reported by Thai women (2.4%) were however lower than corresponding rates reported by Swedish-born women for physical assault (8%) and for sexual coercion (3.2%) in a recent Swedish survey (29). Moreover, most of the Thai women with previously abusive relationships in Thailand were no longer living in abusive relationships in Sweden.

Our findings showing poor mental health in Thai women exposed to IPV in Sweden are in agreement with previous research showing that IPV is strongly associated with poor mental health (1, 2, 15, 16). It may be noted that current exposure but not previous exposure to IPV was significantly related to poor mental health, after adjustment for other factors. IPV and poor mental health are factors, which whether in combination or separately, may lead to marginalization and social isolation, with poor integration as the final outcome, and thus a highly disadvantageous situation for newly arrived Thai women in Sweden. Social isolation was significantly related to IPV in the current study, supporting previous research showing an association between social isolation and IPV, both with IPV as a risk factor for (12) and a consequence (13) of social isolation, among vulnerable groups in society. Raj and Silverman found that socially isolated foreign-born women, especially those without family members other than their partner in the new country, were at risk for severe IPV (12). Thus, socially isolated Thai women may not have the resources to leave potentially threatening partners. Social isolation among abused Thai women in Sweden may increase due to factors such as restricted activity outside the home, fear that reporting IPV might lead to escalation of IPV, lack of knowledge concerning where to seek help, fear of losing the residence permit, and so on. In the study by Morash et al. of immigrant Vietnamese women, abusive men maintained control over the women by limiting the women's work outside the home (30). Thus, the direction of causality between IPV and social isolation may be difficult to determine, especially among foreign-born women.

Social isolation was also strongly related to poor mental health in the current study, thus confirming previous findings indicating that poor mental health and social isolation are strongly interrelated (19). Foreign-born women (vs. native-born women) have increased risks for poorer mental health, including severe mental illness [e.g. (31)]. Moreover, in a Dutch study, increased suicidal behavior among foreign-born women, including South Asians, was due to lack of autonomy and personal freedom (20). For new members of a society, employment and social participation are important ways to reduce the risk for marginalization and/or social exclusion. A study from Australia showed that Thai women married to Anglo-Australian men report social isolation as a main concern (32). Thus, Thai women residing abroad may be at risk for social isolation as well as IPV, due to lack of contact with other close relatives and/or other persons in the surrounding society, and also due to poor language skills. The more than additive effect of social isolation and IPV with regard to poor mental health indicates a vulnerable group of Thai women for whom targeted interventions are particularly needed. Low social participation was only marginally related to IPV and to poor mental health, respectively, as these associations did not reach significance. Very few of the women reported no social participation at all. Moreover, although approximately 50% had two or fewer social activities, it may well be that participation in certain types of activities, such as religious activities with other Thai persons, is sufficiently beneficial in and of itself, such that participation in a greater number of activities would not have had a more beneficial effect on mental health. In contrast, approximately 40% of the women reported social isolation, and social isolation was highly related to poor mental health. The reason for the discrepant results regarding social participation and social isolation is unknown. However, it may well be that, despite a fair amount of participation in various types of social activities, these women perceive themselves as isolated from mainstream Swedish society. The fact that participation in religious activities and private social events were the most frequently reported activities lends indirect support to the notion that these women are mostly socializing within the Thai community. In addition, the effect modification analysis on the effect of IPV and social participation on mental health showed that women with exposure to IPV and high social participation had poorer mental health than women with IPV and low social participation. This may indicate that women in abusive relationships are not getting the support and help they are in need of from their social contacts within the Thai community.

Low social trust was significantly associated with poor mental health, which is in line with previous research (22). Social trust refers to the norms of reciprocity created within the networks people participate in. Social trust is both influenced by previous and current experiences such as IPV, as well as by general norms of trust. Results from the current study show that women with previous exposure to IPV had significantly lower social trust, than women without exposure to IPV. Although social trust may be influenced by previous and/or current experiences in a new country, social trust levels among Thai women may potentially be low due to Thai norms and traditions. Further research would be needed in order to fully explain the origin of the low social trust reported in this group. Finally, the results of the effect modification analysis indicate that social trust, a form of social capital, may have a buffering effect against adverse mental health outcome among abused Thai women. Thus, social trust without exposure to social isolation may lessen the effect of IPV and facilitate integration into the Swedish society.

### Methodological considerations

This is the first study to investigate the prevalence of IPV and its relation to poor mental health among Thai women residing in Sweden. One of the strengths of the study is the relatively large participation rate (62.3%), which is higher than other national or regional public health surveys in 2012 targeting the Swedish-speaking population [e.g. 49%, (33)].

Due to the potential risk that Thai women with IPV might be fearful of responding to the questionnaire, and that such women might risk an exacerbation of an already abusive situation, the questionnaire was distributed in Thai. The majority of the women in our sample are married to Swedish men, who normally do not speak Thai. However, an information letter in both Thai and Swedish was provided, describing the purpose of the study and the contents of the questionnaire without going into detail, to satisfy potential curiosity on the part of the partners. In order to further ensure the safety of the respondents and in accordance with WHO's recommendations (35), the ethical permission from Lund University guaranteed that the methodology was sound and the questionnaire was relevant for the purpose of the project. Also, the code key with names and addresses of the respondents were kept at Statistics Sweden and was inaccessible for the researchers. Finally, an extern Thai-speaking contact person working within the health care sector was attached to the project and could provide support and counseling after the interviews if needed.

However, one-third of the Thai women did not respond to the questionnaire and there is no information on these non-participants; thus, their demographic and socioeconomic background characteristics and possible IPV exposure and mental health status are entirely unknown. Although we lack information on the non-respondents, some of them may not have responded due to a higher proportion of exposure to IPV and poor mental health than respondents. Thus, the current results may well represent an underestimate of the prevalence of IPV in the target population. IPV may also have been underreported among those who participated, due to fear of what might happen if they had reported, both in relation to their partner and in relation to the authorities, as some of them were in a vulnerable situation, having temporary residence permits. Also, IPV might have been underreported due to patriarchal norms and traditions sanctioning IPV and thus leading to a greater tolerance for IPV among Thai women than, for example, among Swedish women. Furthermore, although some measures had missing data, that is, married/cohabitation, educational level, mental health, the proportion of cases with missing data did not exceed 10% for any measure. Finally, for various measures, for example, mental health, summary scores were created and cutoff points determined for within group comparisons. Although the decision as to where to place the cutoff point may have input on the results obtained, cutoffs were constructed based on the examination of the frequency distributions and/or previous validated studies.

This is a unique sample of 804 Thai women residing in two regions in Sweden (Skåne and Sjuhäradsbygden), and the questionnaire was sent out to the entire population of Thai women in the two regions who had arrived since 2006. The questionnaires were distributed by Statistics Sweden, a government organization with registers of all persons with residence in Sweden. Hence, undocumented Thai women and tourists are not part of the study sample. Moreover, the questionnaire was pre-tested in a focus group discussion with a heterogeneous group of Thai women before the final version was completed. An additional strength is that the questionnaire was in Thai, which made it possible to include recently arrived Thai women and also may have facilitated the participation of Thai women with longer residence, who despite better Swedish language skills, may have preferred to respond to a questionnaire in Thai.

Although the target population consisted of all Thai women arriving since 2006 in two different Swedish regions, the extent to which the sample is representative for these regions is limited by the participation rate (62.3%). Despite this limitation, the experiences of these women living in Skåne (a densely populated area) and Sjuhäradsbygden (a smaller and average Swedish region) may generally resemble those of Thai women in other parts of Sweden. However, generalizability of the current results may be limited for Thai women with longer duration of residence in Sweden. Also, the possibility exists that Thai women living in more rural areas (sparsely populated) in, for example, the north of Sweden perceive themselves as even more isolated, and thus the current results may represent an underreporting of social isolation.

A possible limitation is that there is no information on the country of origin of the non-Scandinavian partners, but these were relatively scarce. The majority (75%) of the women who reported country of origin of their partner reported Sweden or another Scandinavian country. An additional limitation is the lack of information concerning the severity and the frequency of IPV, that is, whether it was repeated abuse or a single occurrence. Also, it is unknown whether IPV in Thailand occurred only prior to migration to Sweden or also after, for example, during a vacation in Thailand. The fact that this is a cross-sectional design that prevents any inferences regarding the direction of causality, and there is no way of knowing if mental health problems were present before or after exposure to IPV. Thus, women suffering from poor mental health often are in a more vulnerable situation and may be at greater risk for abuse than women with good mental health (2).

It may be noted that the questions were formulated for use as a postal survey, in contrast to the methodology used in the WHO multi-country study where a structured interview was conducted (14). Thus, the extent to which the current participants fully understood the question is unknown. However, pre-testing of the questions among Thai women did not indicate that the questions pertaining to IPV were regarded as difficult to comprehend or unusually sensitive. Also, the study lacks a comparison group, either Swedish or other foreign-born women, with regard to prevalence rates of IPV. Moreover, other limitations are that physical/sexual IPV and emotional IPV were aggregated into one variable for the purpose of multivariate analysis due to relatively small numbers, and that the sample size may not have been sufficient for detecting differences in the effect modification analyses. Finally, it should be noted that the current study only concerns IPV and that other types of interpersonal violence also may contribute to poor mental health.

Although the GHQ-12 has been used in a wide variety of cultural settings (27), the validity of the instrument among Thai women is relatively unknown. However, the GHQ-12 has previously been used in a study of university students in Thailand (34). Nevertheless, GHQ-12 might not be fully adequate to describe the range and depth of potential mental health consequences of partner abuse.

### Implications and future research

This is the first study investigating the prevalence of IPV and its possible relationship to poor mental health among Thai women residing in Sweden. The results show that most Thai women had been exposed to IPV, and that the IPV prevalence among the women was lower after they had moved to Sweden than when they resided in Thailand. However, a proportion of Thai women residing in Sweden report lifetime exposure to IPV, and those with current IPV exposure report poor mental health, perceived social isolation, and low social trust. The role of social capital in increasing resilience against poor mental health for those living in abusive relationships indicates a need for supporting social structures that facilitate Thai women's’ access to networks outside their own group. Further research is needed in order to fully understand the underlying determinants for exposure to IPV and poor mental health among Thai women in Sweden.
